# Human umbilical cord tissue-derived mesenchymal stromal cells attenuate remodeling after myocardial infarction by proangiogenic, antiapoptotic, and endogenous cell-activation mechanisms

**DOI:** 10.1186/scrt394

**Published:** 2014-01-10

**Authors:** Diana Santos Nascimento, Diogo Mosqueira, Luís Moura Sousa, Mariana Teixeira, Mariana Filipe, Tatiana Pinho Resende, Ana Francisca Araújo, Mariana Valente, Joana Almeida, José Paulo Martins, Jorge Miguel Santos, Rita Nogueira Bárcia, Pedro Cruz, Helder Cruz, Perpétua Pinto-do-Ó

**Affiliations:** 1 INEB, Instituto de Engenharia Biomédica, Universidade of Porto, Rua do Campo Alegre, 823, 4150-180 Porto, Portugal; 2 ICBAS, Instituto de Ciências Biomédicas Abel Salazar, Universidade of Porto, Porto, Portugal; 3 ECBio, Investigação e Desenvolvimento em Biotecnologia, S.A, Rua Henrique Paiva Couceiro, 27, 2700-451 Amadora, Portugal; 4 CICS, Centro de Investigação em Ciências da Saúde, Departamento de Ciências Farmacêuticas, Instituto Superior de Ciências da Saúde, Norte, Paredes, Portugal

## Abstract

**Introduction:**

Among the plethora of cells under investigation to restore a functional myocardium, mesenchymal stromal cells (MSCs) have been granted considerable interest. However, whereas the beneficial effects of bone marrow MSCs (BM-MSCs) in the context of the diseased heart are widely reported, data are still scarce on MSCs from the umbilical cord matrix (UCM-MSCs). Herein we report on the effect of UCM-MSC transplantation to the infarcted murine heart, seconded by the dissection of the molecular mechanisms at play.

**Methods:**

Human umbilical cord tissue-derived MSCs (UCX®), obtained by using a proprietary technology developed by ECBio, were delivered via intramyocardial injection to C57BL/6 females subjected to permanent ligation of the left descending coronary artery. Moreover, medium produced by cultured UCX® preconditioned under normoxia (CM) or hypoxia (CMH) was collected for subsequent *in vitro* assays.

**Results:**

Evaluation of the effects upon intramyocardial transplantation shows that UCX® preserved cardiac function and attenuated cardiac remodeling subsequent to myocardial infarction (MI). UCX® further led to increased capillary density and decreased apoptosis in the injured tissue. *In vitro*, UCX®-conditioned medium displayed (a) proangiogenic activity by promoting the formation of capillary-like structures by human umbilical vein endothelial cells (HUVECs), and (b) antiapoptotic activity in HL-1 cardiomyocytes subjected to hypoxia. Moreover, in adult murine cardiac Sca-1^+^ progenitor cells (CPCs), conditioned medium enhanced mitogenic activity while activating a gene program characteristic of cardiomyogenic differentiation.

**Conclusions:**

UCX® preserve cardiac function after intramyocardial transplantation in a MI murine model. The cardioprotective effects of UCX® were attributed to paracrine mechanisms that appear to enhance angiogenesis, limit the extent of the apoptosis, augment proliferation, and activate a pool of resident CPCs. Overall, these results suggest that UCX® should be considered an alternative cell source when designing new therapeutic approaches to treat MI.

## Introduction

Cardiac diseases, in particular, ischemic heart disease, represent a major cause of mortality and morbidity worldwide, imposing a heavy economic burden on high- and middle-income countries [1]. Myocardial infarction (MI) is characterized by extensive cardiomyocyte death and functional degradation of the cardiac tissue downstream from a coronary occlusion. After infarction, a cascade of dramatic biochemical and morphologic events, collectively designated cardiac remodeling, is initiated. These events culminate in the formation of a nonfunctional scar in place of the damaged myocardium. Currently, no therapeutic solutions are available for replacing the net loss of contractile tissue by new cardiomyocytes. The only efficient long-term treatment is heart transplant, which is impaired mainly by the shortage of organ donors and the requirement for immunosuppression [2]. Consequently, cell therapies aimed at directing heart’s response toward a more-efficient repair and/or myocardial regeneration have received considerable attention in the last decade [3].

The potential for cardiac regeneration or, at the least, for improving the myocardial repair process, is under investigation for a plethora of different adult and embryonic-derived cell populations [4]. Mesenchymal stromal cells (MSCs) have taken special prominence because of their immunomodulatory role, which enables major histocompatibility complex-mismatched allogeneic transplantation [5]. Although originally harvested from the bone marrow [6,7], MSC-like cells have also been identified in different tissues, and the most common sources are adipose tissue [8] and umbilical cord tissue [9]. The use of allogeneic umbilical cord tissue, namely umbilical cord matrix (Wharton's jelly), is advantageous in comparison to any other source or because of (a) the extraction process, i.e. absence of invasiveness and higher efficiency, (b) higher MSCs cell yield [10], and (c) the shorter MSCs doubling time, enabling a faster attainment of the abundant cell numbers required for clinical use [11,12] while maintaining a safe profile.

Furthermore, we recently showed that UCX® were more potent modulators of the immune system than bone marrow-derived MSCs (BM-MSCs), being able to repress T-cell activation and promote the expansion of regulatory T cells more efficiently [13].

It has been extensively demonstrated that BM-MSCs can be harnessed for cardiac repair [14-18], with clinical trials showing promising results [19]. Several preclinical studies have demonstrated the improvement of heart function and the reduction of infarct size after MSCs transplantation [20-25]. The cardioprotective mechanisms exerted by MSCs appear to encompass (a) transdifferentiation/fusion events [26-29], (b) attenuation of cardiac remodeling [20,30-32], and (c) enhancement of angiogenesis [25,33-35]. These appear to occur via cellular engraftment into the host myocardium [36] and/or by a paracrine action (that is, favoring cardiac repair by MSCs-secreted factors) [37]. MSCs are frequently regarded as factories of cytokines and growth factors [38,39] whose production is often increased under hypoxic conditions [35]. The paracrine action of MSCs in the context of MI has been demonstrated by the recapitulation of the cardioprotective effects attained by cell transplantation through administration of the conditioned medium produced *in vitro* by the same cells [40].

Although the beneficial effects of BM-MSCs in the context of the diseased heart have been extensively reported, data are still scarce on the effect of MSCs from the umbilical cord tissue (UCM-MSC) [23,41-44]. Thus, we set out to investigate the effect of transplantation of a well-defined umbilical cord tissue-derived cellular product (UCX®) on the heart of myocardial infarcted mice, seconded by the dissection of the molecular mechanisms at play.

In this study, a specific population of human stem cells derived from the umbilical cord tissue (Wharton’s jelly), hereafter designated UCX®, was isolated, expanded, and cryopreserved on the basis of proprietary technology developed within our team [43]. We show that UCX® delivery into the myocardium of mice subjected to left anterior descending (LAD) coronary artery ligation (a) preserves heart function, (b) attenuates the cardiac remodeling process, (c) increases capillary density, and (d) prevents apoptosis in the infarcted tissue. Moreover, *in vitro,* we demonstrated that UCX® exerts a beneficial effect on different cellular components of the myocardium through paracrine mechanisms. Hence, UCX® protect cardiomyocytes from hypoxia-induced apoptosis, enhance the formation of capillary-like structures by endothelial cells, and trigger the differentiation of Sca-1^+^ adult cardiac progenitor cells (CPCs) [45].

## Material and methods

### Ethics and regulation

This study was approved by the Ethics Committee at the Cascais Hospital Dr. José de Almeida, in the scope of a research protocol between ECBio–Research & Development in Biotechnology, S.A., and HPP Saúde–Parcerias Cascais, S.A. In addition, all experimental research was in compliance with the Helsinki Declaration. Umbilical cord donations were obtained with written informed consents, according to Directive 2004/23/EC, which sets the standards of quality and safety for the donation, procurement, testing, processing, preservation, storage, and distribution of human tissues and cells [46]. All the animal-testing procedures were subjected to approval by the IBMC-INEB (Instituto de Biologia Molecular e Celular–Instituto de Engenharia Biomédica) Animal Ethics Committee, and to the Direcção Geral de Veterinária (permit 022793), and are in conformity with the Directive 2010/63/EU of the European Parliament [47]. Humane end points were followed in accordance to the OECD Guidance Document on the Recognition, Assessment, and Use of Clinical Signs as Humane End points for Experimental Animals Used in Safety Evaluation [48].

### UCX® isolation and *ex vivo* maintenance

Human UCX® were isolated according to [13] and patented proprietary technology [44] developed by ECBio. In brief, fresh human umbilical cords were obtained after term natural or C-section births, transported to the laboratory facilities in a sterile container, and processed within 48 hours. The procedure includes three recovery phases to ensure a high cell yield and high isolation success rates.

Isolated UCX® were cultured (up to P7) in Minimum Essential Medium α (α-MEM; Gibco, Carlsbad, CA, USA), 1% P/S (100 U/ml Penicillin and 100 μg/ml Streptomycin, Labclinics, Barcelona, Spain), buffered with 10 m*M* HEPES (Gibco), hereafter designated Basal Medium (BM), supplemented with 20% Fetal Bovine Serum (FBS; Lonza, Basel, Switzerland), in a humidified incubator at 37°C and 5% CO_2_. Cells at confluence >90% were subcultured by using Tryple Select (Gibco) as a detaching agent.

### Myocardial infarction, UCX® delivery, and echocardiography

Adult C57BL/6 mice (Charles River, Wilmington, MA, USA) aged 8 to 12 weeks, were used for this study, independent of gender. Myocardial infarction was experimentally induced by LAD coronary artery ligation, as previously described [49] with minor alterations. In brief, mice were anesthetized by intraperitoneal injection (ip) of medetomidine (1 mg/kg, Sededorm; ProdivetZN, Lisboa, Portugal) and ketamine (75 mg/kg, Clorketam; Vétoquinol, Lure, France), orally intubated, and mechanically ventilated by using a small-animal respirator (Minivent 845; Harvard Apparatus, Holliston, MA, USA). Animals were kept on warming pads throughout the surgical procedure and until full recovery. Under a stereomicroscope (Leica EZ4; Leica Microsystems, Wetzlar, Germany), the heart was exposed (Ø 5 to 7 mm) via left thoracotomy on the third intercostal space, and the pericardial sac was gently disrupted. The first portion of the LAD coronary artery was visualized below the left atrium, as a pulsating bright red vessel. A nonabsorbable 7.0 suture (Silkam; B. Braun, Melsungen, Germany) was passed under the artery, and the ligation was performed.

After LAD ligation, 2 × 10^5^ UCX® in 0.5% Bovine Serum Albumin (BSA; Merck, Whitehouse Station, NJ, USA)/phosphate-buffered saline (PBS; Gibco) were delivered by four intramyocardial injections of 5 μl each, with a Hamilton syringe (Hamilton, Reno, NV, USA) attached to a 30-gauge needle (*n* = 5). A group of control animals (*n* = 10) were subjected to the same surgical procedure, although injected only with the vehicle (0.5% BSA in PBS). The intercostal incision was closed by an absorbable 6.0 suture (Safil; B. Braun), and surgical staples were used for skin closure. Anesthesia was reversed by 5 mg/kg ip atipamezole (Revertor; Virbac, Carros, France). Analgesia and fluid therapy were performed by ip delivery of butorphanol (1 mg/kg; Butador; Richter Pharma AG, Wels, Austria) and subcutaneous injection of 5% glucose physiologic saline (B. Braun), respectively. This procedure was repeated every 12 hours up to 72 hours after surgery or until full recovery.

Transthoracic echocardiography was performed at 7 and 14 days after LAD coronary artery ligation by using a portable ultrasound apparatus (GE Vivid I; General Electric, Fairfield, CT, USA) equipped with a 12-MHz linear probe (GE 12 L-RS Linear Array Transducer; General Electric). Animals were anesthetized (Clorketam, 100 mg/kg) and placed on left lateral decubitus position. Two-dimensional (2D) mode images of parasternal short-axis view were acquired to position the Motion-mode (M-mode) cursor at the level of the papillary muscles and perpendicularly to the interventricular septum and left ventricle (LV) free-wall. To evaluate LV structural changes, several parameters from M-mode were measured (that is*,* the LV internal diameter at diastole (LVIDd) and at systole (LVIDs). Left ventricular ejection fraction (EF) and fraction shortening (FS) were calculated as an index of systolic function: FS (%) = ((LVIDd - LVIDs)/LVIDd) × 100 and EF (%) = ((LVIDd^3^ – LVIDs^3^)/LVIDd^3^) × 100. The same parameters were measured on control nonmanipulated (np) healthy animals (*n* = 10).

### Histologic procedures and immunohistochemistry

At 14 days after surgery, animals were deeply anesthetized by ip injection of pentobarbital (Eutasil; CEVA, Algés, Portugal, 70 mg/kg). After 4 *M* potassium chloride (Sigma-Aldrich, St. Louis, MO, USA) injection, diastole-arrested hearts were harvested, briefly washed in PBS, and fixed in 10% formalin neutral buffer (Prolabo; VWR International, Radnor, PA, USA) up to 24 hours before paraffin embedding. Representative sampling of the LV (approximately 12 sections, 3 μm) was obtained by transverse sectioning from the apex to the base of paraffin-embedded hearts with an interval of 300 μm between each section. Infarct-size assessment was performed by staining paraffin sections with modified Masson trichrome staining (MT), according to the Trichrome (Masson) Stain kit (Sigma-Aldrich), with the following modifications: nuclei were prestained with Celestine Blue solution after staining with Gill’s Hematoxylin and incubation for 1 hour in aqueous Bouin solution to promote uniform staining.

Immunostaining of heart sections were performed as follows. After antigen recovery, sections were permeabilized with 0.2% Triton X-100 (Sigma-Aldrich) for 5 minutes and incubated 1 hour in 4% FBS/1% BSA in PBS for blocking unspecific staining. For CD31 immunodetection, sections were incubated with primary antibody (sc1506, goat anti-mouse CD31; Santa Cruz Biotechnology, Dallas, TX, USA), diluted 1:400 in the blocking solution, for 2 hours at room temperature (RT). Thereafter, sections were incubated with the secondary antibody (A11057, 568 AlexaFluor donkey anti-goat IgG; Invitrogen) diluted 1:1,000 in blocking solution, for 1 hour at RT. Sections were finally mounted by using Fluoroshield containing DAPI (F6057; Sigma-Aldrich), for nuclei detection.

The extent of apoptosis in the cardiac tissue was assessed by TUNEL assay (ApopTag Fluorescein In Situ Apoptosis Detection Kit; Millipore, Billerica, MA, USA), preceded by antigen recovery with Proteinase K (Sigma-Aldrich; 20 μg/ml, for 15 minutes at 37°C). Images were acquired with the inverted fluorescence microscope Axiovert 200 Motorized and an AxioCam MRm camera (Zeiss, Oberkochen, Germany). Quantification of apoptotic and endothelial cells was performed by using ImageJ 1.42 software (National Institutes of Health (NIH), Bethesda, MD, USA).

### Measurement of myocardial infarct size and morphometric analysis

For infarct-size determination, the collagen deposition highlighted (blue) in MT-stained sections was used to define the LV scarred region. Images of histologic sections were captured with an Olympus SZX10 stereomicroscope and Olympus DP21 camera (Shinjuku, Tokyo, Japan). The percentage of the ischemic LV wall was calculated by using the semiautomated software *MIQuant*[50] by two different methods: the area measurement [49] and the midline length measurement [51].

Morphometric analysis was performed by resorting to Image J software (NIH). Wall thickness was calculated as the average of the distance across the wall of five equidistant points of the ischemic wall. Lumen-area percentage was calculated by the ratio between the lumen delimited by the endocardium and the total LV area.

### Conditioned medium collection and processing

UCX® was seeded in BM supplemented with 5% FBS and grown until 90% confluence. After washing cells with PBS, BM (without serum) was added, and 24 hours preconditioning under normoxia (37°C, 5% CO_2_) or hypoxia (37°C, 5% CO_2_, 1% O_2_) was performed. Thereafter, BM was replaced and conditioned for 48 hours at 37°C and 5% CO_2_, after which it was collected, centrifuged at 300 *g* for 10 minutes to remove cell debris, filtered (0.22-μm pore size, Millipore), and concentrated in 5-kDa cut-off spin concentrators (Agilent Technologies, Santa Clara, CA, USA). Conditioned medium produced by UCX® subjected to normoxia (CMN) or hypoxia (CMH) was frozen at −80°C for further use.

### Apoptosis assay

The cardiomyocytic cell line HL-1 was maintained in Claycomb supplemented medium (SAFC Biosciences, Lenexa, KS, USA), according to authors instructions (please refer to [52] for details). HL-1 cells were seeded (50,000 cells/cm^2^) on Ø 5-mm coverslips (Thermo Scientific, Waltham, MA, USA) overnight. Immediately before subjecting HL-1 to hypoxia (1% O_2_), medium was changed to Claycomb basal medium supplemented with either (a) α-MEM basal medium (BM); (b) concentrated CMN, (c) concentrated CMH (ninefold dilution), or (d) BM + 10% FBS. After 48 hours of hypoxia (*n* = 3), HL-1 cells were fixed in 1% paraformaldehyde (PFA; Sigma-Aldrich) in PBS (15 minutes, RT), and the percentage of apoptotic cells was determined by using the ApopTag kit (Millipore) followed by image acquisition by the inverted fluorescence microscope Axiovert 200 (Zeiss).

### Vasculogenesis assay

The vasculogenesis assay was performed as described in [53], by using the thick gel method of preparation. In brief, Matrigel growth factor reduced (BD Biosciences, San Jose, CA, USA) was thawed overnight and poured carefully into eight-well chamber slide LabTeks (Nalgene Nunc, Thermo Scientific; 10^5^ μl/well), followed by incubation at 37°C for 45 minutes to allow jellification. Afterwards, human umbilical vein endothelial cells (HUVECs; Science Cell 8000; Corte Del Cero, Carlsbad, CA, USA) up to P10 were cultured (45,000/cm^2^) on top of Matrigel in Endothelial Basal Medium-2 (EBM-2; Lonza) + 1% P/S supplemented (1:9 dilution) with (a) α- MEM basal medium (BM), conditioned medium produced by UCX® subjected to (b) normoxia (CMN) or (c) hypoxia (CMH) preconditioning. After incubation at 37°C and 5% CO^2^ for 3.5 hours, cells were washed in PBS and stained with Calcein AM (C1430; Invitrogen) Images were acquired by using the inverted fluorescence microscope Axiovert 200 (Zeiss). Tube formation, number of branching points, and tube length and thickness were manually measured by using ImageJ1.42 (NIH), analyzing approximately 25 fields per replicate (*n* = 3).

### Isolation and culture of adult murine heart Sca-1^+^ cardiac progenitor cells (CPCs)

Sca-1^+^ CPCs were extracted and selected by immunomagnetic cell sorting (MACS; Miltenyi Biotec, Bergisch Gladbach, Germany). In brief, hearts from six adult C57BL/6 mice were harvested, dissociated by using the GentleMACS Dissociator (Miltenyi Biotec), and digested with 600 U/ml Collagenase II (4176; Worthington, Lakewood, NJ, USA) and 60 U/ml DNAseI (A3778; Applichem Lifesciences, Gatersleben, Germany) for 30 minutes at 37°C with agitation (100 rpm). Immunomagnetic cell sorting was performed by incubating the single-cell suspension with Sca-1-FITC antibody (MACS; Miltenyi Biotec) followed by anti-FITC Microbeads (MACS; Miltenyi Biotec). Sca-1^+^ CPCs were maintained in α-MEM basal medium (BM) supplemented with 10% FBS. Cells were split at 90% confluence and used until passage 3.

### Sca-1^+^ CPCs metabolic activity, proliferation assessment, and quantitative real-time PCR (qRT-PCR)

Sca-1^+^ CPCs were trypsinized and seeded in four different media: (a) α-MEM basal medium (BM); (b) basal medium supplemented with 10% FBS (BM + FBS); (c) CMN; or (d) CMH. After 72 hours of culture, media were replaced by 10% resazurin (R7017; Sigma-Aldrich) in BM and incubated for 3 hours, after which absorbance at 590 nm was measured. Metabolic activity values are expressed relative to BM treatment for each initial cell-plating density.

Proliferation was assessed by Ki67 immunostaining in CPCs fixed in 4% PFA and permeabilized with 0.2% Triton-X for 2 minutes. After blocking with 4% FBS/1% BSA in PBS for 1 hour, cells were incubated with anti-Ki67 primary antibody (1:200, ab15580; Abcam, Cambridge, England) overnight at 4°C and subsequently with the secondary antibody Alexa Fluor 568 donkey anti-rabbit (1:300, A-10042; Invitrogen), for 2 hours at RT. Images were acquired by the inverted fluorescence microscope Axiovert 200 (Zeiss), and percentage of Ki67^+^ cells was manually determined by counting approximately five fields with 30 to 50 cells per field, per replicate and medium condition.

For qRT-PCR, RNA was extracted by using RNAeasy extraction kit (Qiagen, Hilden, Germany) and reverse transcribed by using PrimeScriptRT reagent kit (RR037A; Takara, Tokyo, Japan). PCR was performed by using the iQSYBR Green SuperMIX (BioRad, Hercules, CA, USA) and according to the iQ5 Real-Time PCR Detection System (BioRad). Primer sequences and temperature cycles will be available on request. mRNA expression was defined as primer efficiency to the power of the difference in threshold cycle values between the gene of reference (*GADPH*) and the gene of interest. Values were expressed relative to those derived from CPCs cultured in standard conditions (BM + 10% FBS).

### Data and statistical analysis

Values presented in text and figures are as mean ± standard error of the mean of at least three independent experiments. Statistical analysis was performed by using Graph Pad Prism (v5, La Jolla, CA, USA) software, evaluated by unpaired one-way ANOVA test by using Newman-Keuls *post hoc* test for correction of multiple comparisons, and differences were considered significant when *P < 0.05, **P < 0.01, or ***P < 0.0001.

## Results

### Intramyocardial transplantation of UCX® preserves cardiac performance and attenuates adverse LV remodeling 2 weeks after MI

UCX® were isolated by using a proprietary method (PCT/IB2008/054067) that yields a well-defined number of cells by using a precise proportion between tissue-digestion enzyme activity units, tissue mass, digestion solution volume, and void volume. These cells displayed plastic adherence, fibroblast-like morphology, and an antigenic profile typical of mesenchymal stromal cells [13] (see Additional file 1: Figure S1), as defined by the International Society for Cellular Therapy [54].

**Figure 1:**
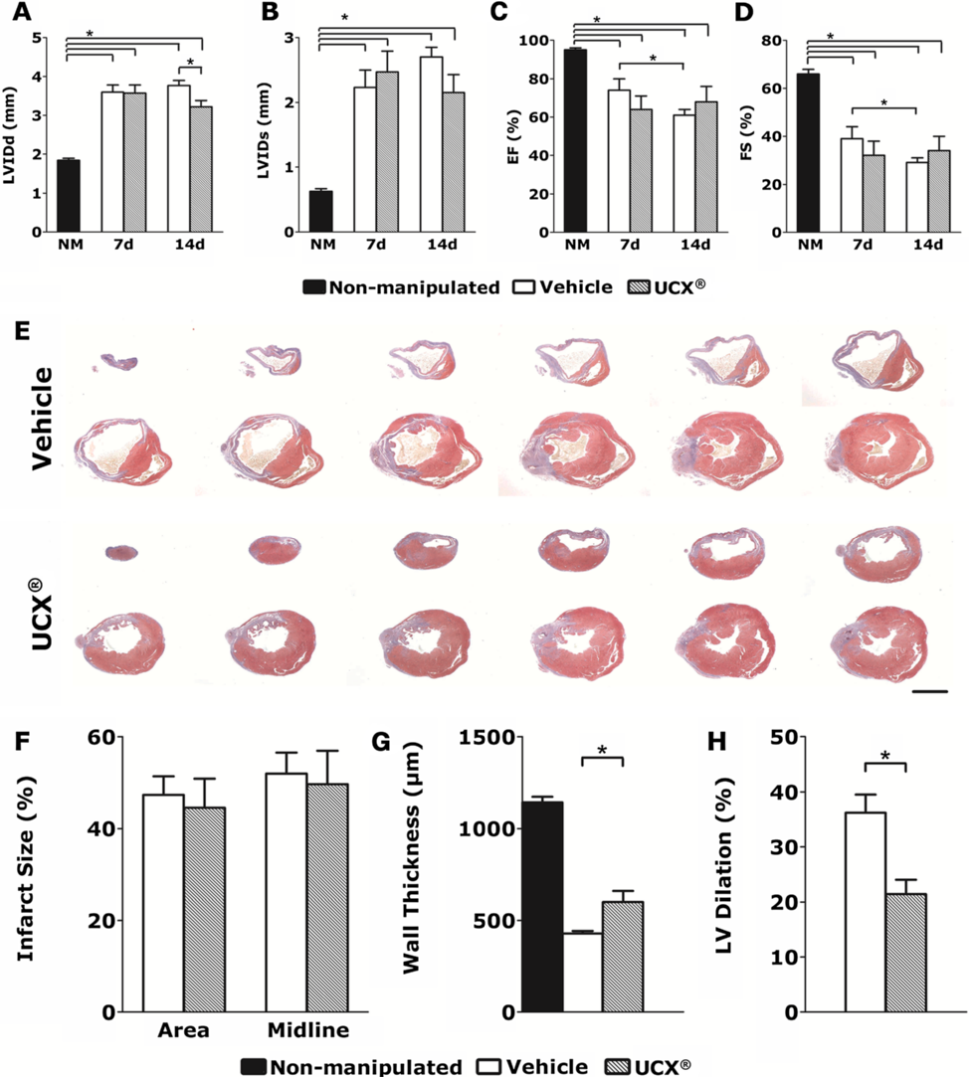
**Intramyocardial delivery of UCX® preserved cardiac function and attenuated cardiac remodeling after MI. (A, B)** Transthoracic echocardiography, at 2 weeks after transplantation, demonstrated a significant reduction in diastolic LVID and a more subtle reduction in systolic LVID in the UCX® transplanted group (*n* = 5) when compared with vehicle-injected animals (*n* = 10). **(C, D)** EF and FS of UCX®-transplanted hearts was preserved from 1 to 2 weeks after injury, contrarily to the progressive decrease of the systolic function observed in the vehicle-injected group. **(E)** Masson Trichrome stain on histologic sections representative of the LV highlight the collagen deposition (blue) on the infarcted region. Thinning of the LV wall and LV dilatation was observed in the UCX® (*n* = 5), as well as in the vehicle (*n* = 10) experimental groups. **(F)** MI size was quantified by two different methods: the area measurement and the midline length measurement*,* and no significant differences were identified between groups. When compared with the vehicle control group the UCX®-transplanted group showed a smaller reduction of the LV wall thickness **(G)** and a less-pronounced LV dilation **(H)**, indicative of UCX®-mediated tapering of cardiac remodeling after MI. LVIDd, left ventricular internal diameter at diastole; LVIDs, left ventricular internal diameter at systole; EF, ejection fraction; FS, fractional shortening; *n* (nonmanipulated) = 10. *P < 0.05; scale bar = 2 mm.

To evaluate the therapeutic potential of the UCX® in an MI setting, cells were delivered via intramyocardial injection to mice subjected to permanent ligation of the LAD coronary artery. Infarcted animals that received only the vehicle were used as control. Transthoracic echocardiography performed at 1 and 2 weeks after MI revealed consistent LV-chamber dilation and impaired LV function in all MI groups (Figure 1A through D). Although no beneficial effect of UCX® delivery could be recognized at 1 week after MI, a tendency to decreased EF and FS in transplanted hearts was evident (Figure 1C, D). Conversely, at 2 weeks after MI, a significantly reduced diastolic LV-chamber dilation could be verified in cell-transplanted mice when compared with the vehicle control group (Figure 1A, B). Moreover, although not reaching statistical significance, EF and FS had inverted the tendency observed at 1 week and were augmented in the cell-transplanted group when compared with vehicle-injected hearts (Figure 1C, D). Overall, UCX® transplanted hearts preserved systolic function 2 weeks after surgery, contrarily to the progressive loss of cardiac function observed in the vehicle group.

Widespread cell death and degradation of the cardiac extracellular matrix in the ischemic myocardium culminates in a substantial thinning of the LV wall, LV chamber dilation, and formation of a collagenous-rich nonfunctional scar; alterations that are commonly designated as cardiac remodeling. Stereoscopic view of representative cross-sections of infarcted hearts harvested at 2 weeks after surgery showed that LV free-wall thinning and LV chamber dilation occurred in both experimental groups (Figure 1E). Morphometric analysis of the LV cross-sections revealed that UCX® transplanted hearts exhibited a less-pronounced decrease (by 41.1% ± 13.8%) in thickness of infarcted LV wall (Figure 1G) than the vehicle control group. Moreover, the UCX®-injected group displayed a 40.8% ± 7.2% reduction in the endocardial diameter, relative to the vehicle-control (Figure 1H), similar to what was observed in the echocardiographic assessment of the LVIDd (Figure 1A). These data suggest an UCX®-mediated cardiac protective effect through attenuation of the infarct expansion and LV remodeling after MI.

Despite these evidences, no differences in infarct size (calculated on the basis of collagen deposition) were observed between vehicle and UCX® transplanted MI animals (Figure 1F).

### UCX® cells stimulate angiogenesis and prevent apoptosis in the infarcted myocardium

**Figure 2:**
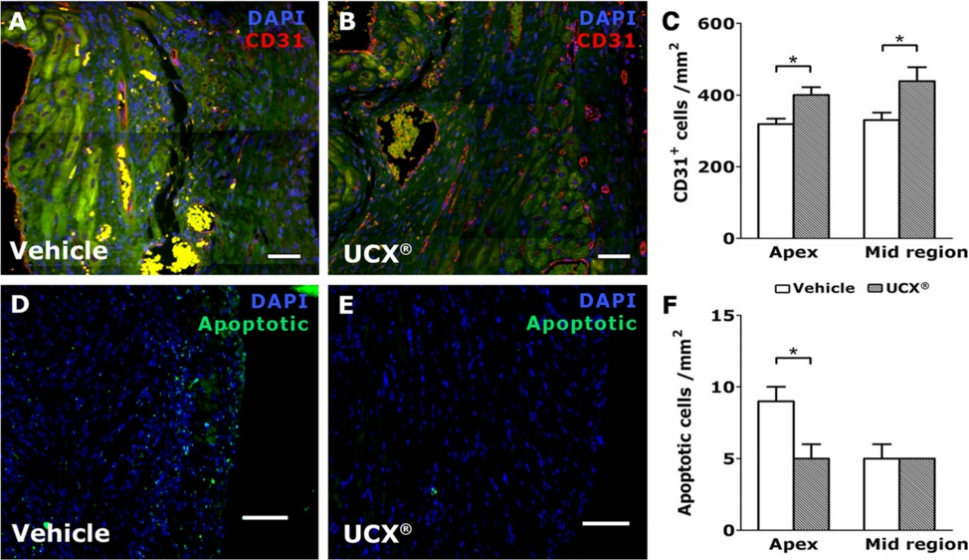
**Transplantation of UCX® into MI hearts increased capillary density and decreased apoptosis.** Representative images of vehicle–injected **(***n =* 6, **A** and **D)** and UCX®-transplanted hearts **(***n =* 5*,***B** and **E)** stained for CD31 **(A** and **B**, red**)** and for cellular apoptosis **(D** and **E**, bright green). **(C)** CD31-expressing cells were more abundant within the infarcted LV wall of UCX®-transplanted hearts when compared with the vehicle control group. **(F)** UCX®-transplanted hearts showed fewer apoptotic cells in the apical region when compared with vehicle-injected hearts, suggesting an anti-apoptotic protective effect by the UCX®. Green filter in **A** and **B** denotes tissue autofluorescence. *P < 0.05; scale bars = 100 μm.

To evaluate whether the cardiac improvement observed after MI could be in part a result of improved neovascularization, CD31-expressing cells (CD31^+^) were immunolabeled and counted in the infarcted and peri-infarcted regions of all experimental groups. Quantification of CD31^+^ cells demonstrated a clear increase in the number of endothelial cells of the microvasculature on UCX®-transplanted hearts (Figure 2B) over the vehicle group (Figure 2A). This increase was more pronounced in the myocardium adjacent to the ligation region (32.9% ± 7.8%) than in the apex region (25.3% ± 11.8%) of the LV (Figure 2C).

Moreover, the anti-apoptotic potential of UCX® was evaluated on the infarcted and peri-infarcted regions (Figure 2D and E). The number of apoptotic cells in the apex of UCX®-transplanted hearts was reduced by 39.0% ± 8.7% when compared with the same region of vehicle-injected hearts (Figure 2F).

Although a beneficial effect of UCX® in cardiac repair following MI could be established, it remained to be elucidated whether functional and histologic improvement was a direct result of UCX® engraftment in the injured hearts. To assess this, paraffin-embedded heart tissue sections were stained with a monoclonal antibody directed against the nuclei of human cells. Nuclear-specific staining could not be found (see Additional file 1: Figure S2), indicating that UCX® did not engraft the murine heart in this experimental setting. Human muscle tissue subjected to similar processing was used as positive control.

### UCX® enhances cellular vasculogenesis *in vitro* via a paracrine mechanism

**Figure 3:**
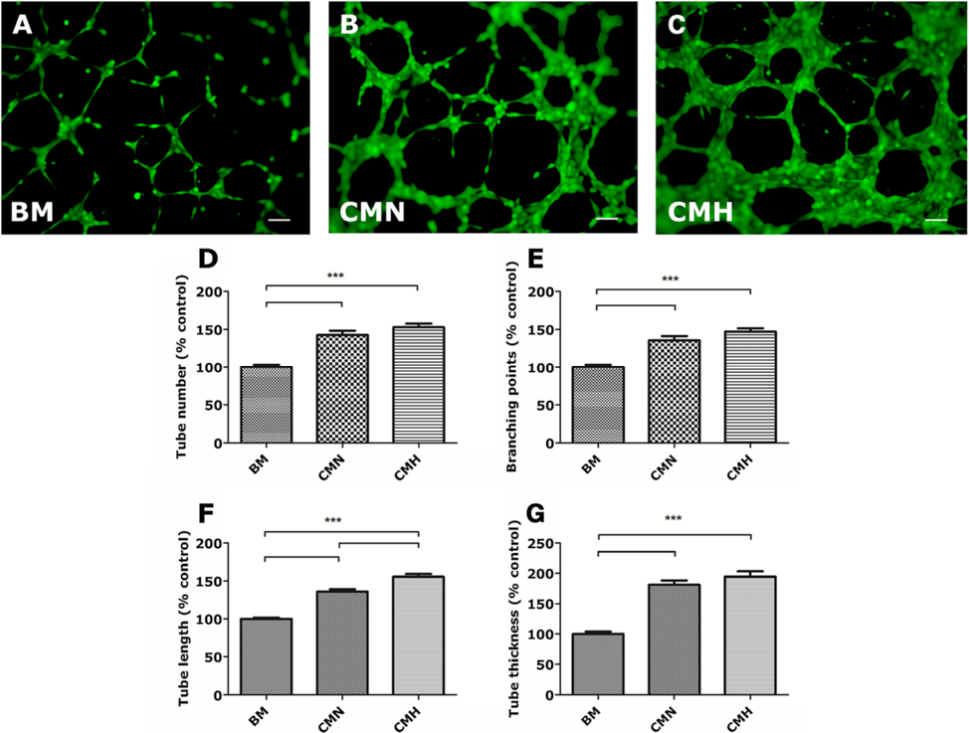
**Paracrine effect of UCX® in *in vitro *vasculogenesis by HUVECs.** Matrigel assay was performed by seeding HUVECs in EBM-2 supplemented with BM **(A)**, CMN **(B)** or CMH **(C)** for 3,5 hours. Cells seeded in UCX®-conditioned media formed more capillary-like structures **(D**, by 42.7% ± 5.6% in CMN and 53.1% ± 4.7% in CMH) and branching points **(E**, by 35.8% ± 5.6% in CMN and 47.4% ± 4.3% in CMH), suggesting a pro-angiogenic effect. The tubes formed in CMN- or CMH-supplemented media were longer **(F**, by 35.8% ± 3.2% in CMN and 55.5% ± 3.7% in CMH) and thicker **(G**, by 81.4% ± 6.8% in CMN and 94.8% ± 8.8% in CMH), in comparison to those formed in BM, further corroborating the observed *in vivo* vasculogenic effect. ***P < 0.0001.

Given that UCX® did not engraft murine hearts, we postulated that the observed cardioprotective effects could have been exerted primarily through paracrine mechanisms. Thus, conditioned medium produced by UCX® subjected to either normoxia (CMN) or hypoxia (CMH) preconditioning was collected for further investigation. Because UCX® enhanced the number of cells expressing the prototypical endothelial marker CD31 in MI-hearts *in vivo*, we addressed whether the UCX®-conditioned media were able to enhance the formation of capillary-like structures by endothelial cells (HUVECs) *in vitro*, by using a classic vasculogenesis assay (Matrigel). In comparison with HUVECs seeded in endothelial basal medium (EBM-2) supplemented with BM (Figure 3A), cells cultured in CMN (Figure 3B) or CMH-supplemented media (Figure 3C) showed an increased number of capillary-like structures (by 42.7% ± 5.6% in CMN and 53.1% ± 4.7% in CMH, Figure 3D), as well as branching points (by 35.8% ± 5.6% in CMN and 47.4% ± 4.3% in CMH, Figure 3E). The caliber of the tubes formed was also affected, as HUVECs cultured in CMN- or CMH-supplemented EBM-2 displayed lengthier (by 35.8% ± 3.2% in CMN and 55.5% ± 3.7% in CMH, Figure 3F) and thicker (by 81.4% ± 6.8% in CMN and 94.8% ± 8.8% in CMH, Figure 3G) capillary-like structures, when compared with BM-supplemented medium. These results strongly support that UCX® favors angiogenic processes by a paracrine mechanism, thus corroborating the aforementioned *in vivo* observations (Figure 2A through C).

### UCX® display a paracrine protective effect against the apoptotic damage of HL-1 cardiomyocytes *in vitro*

The UCX®-mediated apoptotic protective effect detected in transplanted MI hearts (Figure 2D through F) led us to investigate whether UCX®-conditioned medium recapitulated such effects *in vitro*.

For this purpose, the HL-1 cell line, a gold-standard system for investigating cardiomyocytes *in vitro*, was used. These cells are able to maintain a differentiated cardiac phenotype while beating in culture.

**Figure 4:**
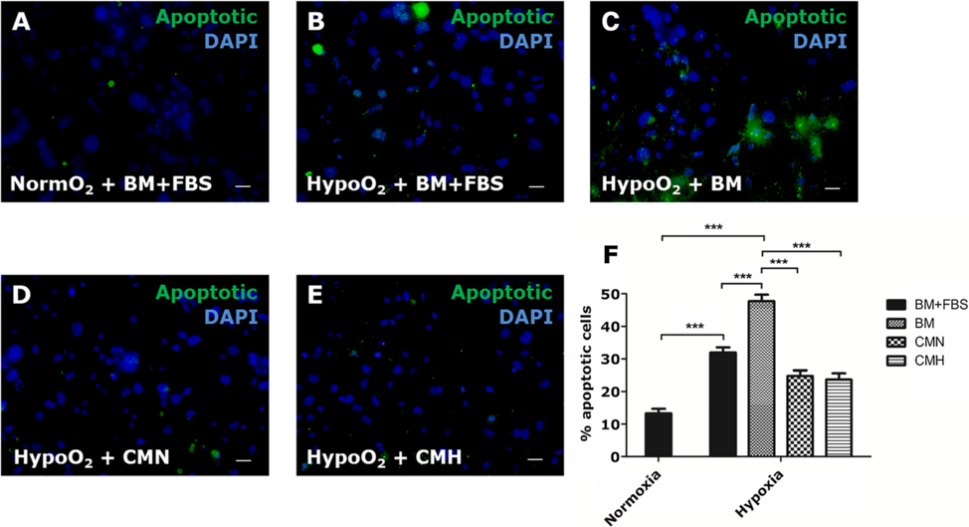
**Paracrine effect of UCX® in hypoxia-induced apoptosis of HL-1 cardiomyocytes. (A through E)** UCX®-derived CMN or CMH protect HL-1 cardiomyocytes from apoptosis (induced by hypoxia during 48 hours), as shown by the detection of apoptotic cells by TUNEL assay. **(F)** The percentage of apoptotic cells increases when cells are subjected to hypoxia for 48 hours, both in serum-containing (31.93% ± 1.59%) and serum-free BM medium (47.73% ± 2.02%). Importantly, when media are supplemented with UCX® conditioned media, the percentage of apoptotic cells is significantly decreased (CMN – 24.77% ± 1.70%; CMH – 23.67% ± 1.95%). ***P < 0.0001, *n* = 4.

HL-1 cells subjected to hypoxic conditions for 48 hours increased the apoptotic rate by approximately 2.4-fold in serum-supplemented medium and to 3.6-fold in serum-free medium (Figure 4A through C). UCX®-CMN and CMH significantly halved the number of TUNEL-positive cells when compared with BM (Figure 4D through F). No significant differences were found in the protection against the hypoxia-induced HL-1 apoptotic damaged between CMN and CMH.

### UCX®-derived conditioned media stimulate resident CPCs proliferation and activate a cardiomyogenic gene-expression program

**Figure 5:**
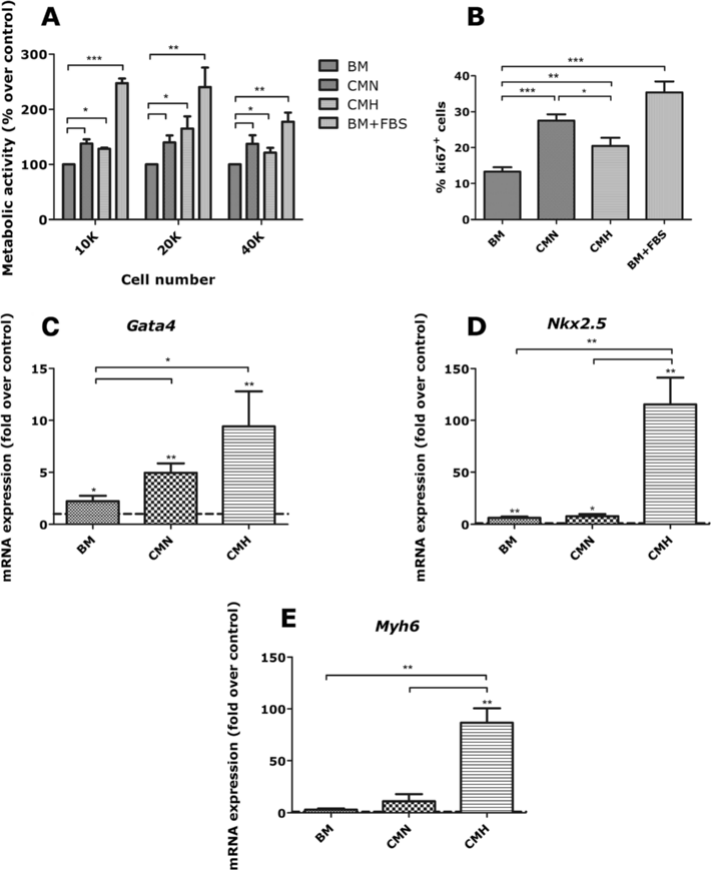
**Paracrine effect of UCX® on CPCs proliferation and cardiac commitment.** Treatment of Sca-1^+^ CPCs with UCX®-derived conditioned media for 72 hours results in enhanced metabolic activity **(A)** and cellular proliferation **(B)**, when compared with the treatment with basal medium (*n* = 4; *P < 0.05; **P < 0.01; ***P < 0.0001). **(C through E)** Treatment with UCX®-derived CMH and, to a lesser extent, CMN triggers CPCs cardiac commitment, as shown by higher expression of early (*Gata4*, *Nkx2.5*) and late (*Myh6*) cardiac-affiliated markers in comparison with basal-medium treated CPCs (*n* = 3; values were normalized to those of standard culture conditions: BM + 10% FBS, represented by the dotted lines in each graph. *P < 0.05; **P < 0.01, either between groups indicated in brackets, or between BM + 10% FBS and the group represented by the asterisk placed above its corresponding column).

Whereas the reportedly low proliferation of mature cardiomyocytes [55] makes it an unlikely event to be accomplished in the time frame of our experimental *in vivo* setting, a stimulation and/or activation of the myocardium undifferentiated cells cannot be disregarded. Therefore, to further dissect the mechanisms involved in UCX®-mediated beneficial effects in MI, we addressed whether resident CPCs could be responsive to UCX® conditioning. Sca-1^+^ CPCs were maintained in either BM, BM-supplemented with FBS, UCX®-CMN, and UCX®-CMH for 72 hours. Although the treatment with both UCX®-conditioned media favored CPCs metabolic activity (Figure 5A) relative to basal culture medium, a differential effect was observed between CMN and CMH concerning CPCs proliferation and commitment. Hence, although both CMN and CMH enhanced the proliferation of Sca-1^+^ CPCs, this effect was more pronounced in response to CMN (Figure 5B). Moreover, higher expression levels of early (*Gata4*, *Nkx2.5*) and late (α heavy chain subunit of cardiac myosin, *Myh6*) cardiac markers revealed that the UCX®-CMH triggers Sca-1^+^ CPCs cardiomyogenic commitment, when compared with the other experimental groups (Figure 5C through G). CMN also induced the expression by CPCs of *Gata-4* and *Myh6*, however, not attaining the levels observed with CMH. Importantly, the results displayed in Figure 5 were verified for three independent experiments (each performed in duplicate) with different batches of conditioned media as well as different preparations of primary cells.

## Discussion

Previous studies have demonstrated that BM-MSCs transplantation has the potential to improve cardiac function and LV remodeling after infarction [56-60]. However, in clinical terms, the use of BM-MSCs presents some severe limitations: (a) the procurement process requires an invasive surgical procedure, (b) the BM-MSCs frequency in the native tissue is very low (1:10,000 total cells in bone marrow), resulting in heterogeneous BM-MSCs preparations that are difficult to replicate; (c) the efficiency of BM-MSCs isolation is also highly dependent on the patient’s clinical history and on the adopted isolation process, and finally, (d) BM-MSCs isolation, in a clinical setting, involves complex logistics and specialized clinicians, which very seldom occur concomitant with common hospital environments [12,61]. In the present work, MSCs isolated from the umbilical cord Wharton’s jelly using technology developed by us, and designated UCX®, come forth as an allogeneic, off-the-shelf cell-product that can be further regarded as the active substance for advanced therapeutic options towards restoration of the injured myocardium. In this MI murine model, UCX® transplantation preserves cardiac function after MI through paracrine mechanism(s) that involve the attenuation of the LV remodeling process and anti-apoptotic as well as pro-angiogenic effects. Potential mechanisms involved in the UCX®-mediated cardiac protection were further dissected *in vitro* by the use of UCX® conditioned media. UCX® paracrine effects involve the (a) protection of cardiomyocytes from hypoxia-induced apoptosis, (b) increase in formation of capillary-like structures by endothelial cells, and (c) promotion of resident Sca-1^+^ CPCs proliferation and cardiac commitment.

Although a clear definition of the mechanisms involved in the use of MSCs as therapeutic agents for MI is not available at this point, a panoply of MSCs-mediated beneficial effects on cardiac function have been reported (apoptosis, scar formation, and angiogenesis) by using different cell sources (for example, bone marrow, adipose tissue) and animal models (for example, rat, pig). In this work, we showed that UCX® transplantation precludes a worsened prognosis of MI over time, contributing to the preservation of cardiac function (% EF and % FS). Moreover, histologic beneficial outcomes were shown on UCX® transplantation, such as an increase in LV wall thickness and lower LV dilation, as reported by others [21,24,25]. Likewise, our data corroborates that a benefic effect of MSCs occurs through the enhancement of angiogenesis [33] and inhibition of apoptosis [62].

Furthermore, a novel aspect in this work is the indication of alternative mechanisms that could account for the cardioprotective activity of UCX® (that is, identification of a potential to activate the cardiac commitment/differentiation and/or augment the frequency of undifferentiated Sca-1^+^ CPCs). In this regard, hypoxia preconditioning of UCX® may play an important role in the determination of cell-fate choice and thereby the function of CPCs. Although both UCX®-conditioned media preparations enhanced CPCs metabolic activity, distinct actions were also evident. Thus, CMN displayed higher mitogenic activity, whereas CMH had a more pronounced effect in the activation of Sca-1^+^ CPCs cardiac commitment, as shown by the higher levels of *Nkx2.5*, *Gata4.* and *Myh6* expression observed in this condition. To the best of our knowledge, this is the first report on the paracrine effects of MSCs in the pool of adult resident undifferentiated cells. Moreover, BM-MSCs have already been shown to affect the differentiation of progenitors isolated from the neonatal heart [63]. Hence, it will be of importance to establish whether the MSCs-mediated effects on CPCs observed *in vitro* correlate with the benefits shown after MSCs transplantation in the onset of MI.

Our study expands the knowledge on the yet-unexplored potential of MSCs derived from umbilical cord tissue to repair the injured heart. Although much work has focused on the properties of MSCs of several distinct sources, only one other study has addressed the umbilical cord tissue-derived cells [23]. The latter reports on transplantation of MSCs in a rabbit MI model as leading to a modest functional recovery of the LV in chronic conditions (30 days after MI), but not in the acute phase (5 days after MI). The cardioprotective effect was attributed to moderate engraftment of the xenotransplanted cells, which expressed immature cardiac markers, despite not being able to differentiate fully into functional cardiomyocytes.

Thus, while corroborating such data, we report on the benefits of UCM-MSCs transplantation in cardiac function after MI in a murine model, and provide additional mechanistic insight into the cardioprotective effects observed.

The UCX® were not found to engraft the murine heart in our setting. Nonetheless, despite the high immunologic barrier to xenotransplantation, adding to the hostile inflammatory *milieu* triggered by MI, and the low dose of administered cells (that is, 2 × 10^5^ cells/heart), the UCX® exerted a beneficial effect in murine infarcted hearts. Although not addressed in the current study, the UCX® likely interfered with the initial events of acute inflammation characteristic of MI, therefore attenuating LV-remodeling processes, while also favoring angiogenic and cardioprotective effects.

Due the well-acknowledged immunomodulatory nature of MSCs, and in particular, that of the MSCs isolated from the umbilical cord tissue [13], persistence of cells in a human allogeneic transplantation may be conceivably higher, thereby enhancing the cardioprotective effects mediated by the paracrine action herein described. This must be considered when designing new cell therapies (that is, cells could be either transplanted to assure prolonged release of factors [64], or alternatively, could be used for producing factors for subsequent administration to the patient.

Administration of UCX®-conditioned medium into the MI border zone may clarify whether paracrine factors solely are responsible for the effects after UCX®-transplantation. Noteworthy, conditioning by the host tissue of the UCX® after transplantation should not be neglected, as it may lead to the production of paracrine factors that are unlikely replicated by the infusion of conditioned media derived from *in vitro* cultured cells. Future work will be conducted to elucidate this particular aspect.

Aiming at a clear indication of which factors could account for the UCX® mediated beneficial effects, the gene-expression pattern of UCX® was assessed with Affimetrix gene array [GEO:GSE51869] by using cells derived from three different umbilical cords (see Additional file 1: Figure S3). When comparing the expression of those genes with that of positive (CD105, CD73, CD90, and CD44) and negative (CD14, CD19, CD34, CD45, CD31, and HLA-DR) markers of UCX®, it is concluded that the expression of angiogenesis-associated transcripts (subtypes of VEGF, angiopoietins, HGF, C-mET, bFGF, TGF-β, and PDGF-AB) is high. Therefore, several factors are most likely contributing for the cardioprotective effects observed *in vivo* and *in vitro*. More-directed work is already under way specifically to identify the role of each of the candidates. Nevertheless, the specific function of these factors in cardiomyocytes protection, vasculogenesis, and CPC induction must be further investigated for clarification of each of these three mechanisms separately.

Yet another approach to enhance cellular paracrine action is to engineer cells to promote their cardioprotective activity. This has been clearly demonstrated in the work by Mangi and colleagues [65]. By genetically engineering rat BM-MSCs to overexpress the prosurvival *Akt-1* gene, a dramatic contribution for the restoration of cardiac function (evaluated *ex vivo*), and prevention of adverse tissue remodeling, was obtained by intramyocardial transplantation after MI.

Noteworthy, the method of UCX® extraction and subsequent processing has been recently adapted to advanced therapy medicinal product (ATMP) standards, as defined by the guideline on the minimum-quality data for certification of ATMP (EMA/CAT/486831/2008/corr, 2010). Given that our effects UCX® exert in the context of MI, a future clinical usage of this off-the-shelf cellular product can be envisaged.

## Conclusions

Human mesenchymal stromal cells derived from the umbilical cord matrix (UCX®) preserve cardiac function and attenuate adverse tissue remodeling after intramyocardial transplantation in a murine MI model. The cardioprotective effect of UCX® is exerted through paracrine mechanism(s) that appear to (a) enhance angiogenesis, (b) limit the extent of the apoptosis in the heart, and (c) to increase proliferation and activate the cardiomyogenic gene-expression program in a pool of myocardial resident cardiac progenitor cells.

## Abbreviations

BM: basal medium; BM + FBS: basal medium supplemented with 10% fetal bovine serum; BM-MSCs: bone marrow-derived MSCs; BSA: bovine serum albumin; CMH: conditioned medium produced after hypoxia preconditioning; CMN: conditioned medium produced after normoxia preconditioning; CPCs: cardiac stem/progenitor cells; EF: ejection fraction; FS: fractional shortening; LAD: left anterior descending; LV: left ventricle; LVIDd: left ventricle internal diameter at diastole; LVIDs: left ventricle internal diameter at systole; MI: myocardial infarction; MSC: mesenchymal stromal cell; MT: Masson trichrome; PBS: phosphate-buffered saline; RT: room temperature; UCM-MSC: umbilical cord matrix-derived MSC.

## Competing interests

The INEB authors have no conflicts of interest to disclose. PC and HC are shareholders and JPM, RNB, JA, MF, MT, and JMS are currently employed at ECBio S.A., a company currently developing cellular therapeutics based on human mesenchymal stromal cells.

## Authors’ contributions

DSN carried out the *in vivo* experiments, participated in the conception of the study, was involved in the experimental conception and design, data analysis and interpretation, and manuscript writing; DM performed *in vitro* experiments with conditioned media, was involved in the experimental conception and design, data analysis and interpretation, and manuscript writing; LMS quantified apoptosis and neovascularization on tissue sections, participated on data analysis and interpretation; MT was involved in the isolation and characterization of UCX® as well as in data analysis and interpretation; MF was involved in the isolation and characterization of UCX® as well as in data analysis and interpretation; MV participated in the animal surgeries and organ collection and was involved in data analysis and interpretation; TPR performed and analyzed the Matrigel assay, and was involved in the critical revising of the manuscript; AFA participated in the immunohistologic characterization and analysis of tissue sections; JA participated in the experimental conception and design and in the critical revising of the manuscript. JPM was involved in the generation and characterization of UCX® and data interpretation and analysis; JMS was involved in the conception of the study, experimental conception and design, and in the critical revising of the manuscript; RNB was involved in the conception of the study, experimental conception and design, and in the critical revising of the manuscript; PC was involved in the conception of the study, experimental conception and design, and in the critical revising of the manuscript. HC was involved in the conception of the study, experimental conception and design, and in the critical revising of the manuscript; PP-O was involved in the conception of the study, coordinated and designed the experimental work, and participated in the manuscript writing. All authors read and approved the final manuscript.

## Supplementary Material

Additional file 1 **The supplementary material includes three additional Figures (Figure S1, Figure S2, and Figure S3) with additional results.** The file also includes the respective legend captions and methods. Click here for file
