# Supplementary material for: Human umbilical cord tissue-derived mesenchymal stromal cells attenuate remodeling after myocardial infarction by proangiogenic, antiapoptotic, and endogenous cell-activation mechanisms
Source: Stem Cell Res Ther. 2014 Jan 10;5(1):5. doi: 10.1186/scrt394 (PMC4055157; doi:10.1186/scrt394)
Supplement: Additional file 1 — The supplementary material includes three additional Figures (Figure S1, Figure S2, and Figure S3) with additional results. The file also includes the respective legend captions and methods. [file scrt394-S1.pdf]

Supplementary materials

Human umbilical cord tissue-derived MSCs (UCX®) attenuate remodeling following myocardial infarction by pro-angiogenic, anti-apoptotic and endogenous cell activation mechanisms

D. S. Nascimento[1,¶], D. Mosqueira[1,¶], L. M. Sousa[1,2,], M. Teixeira[3], M. Filipe[3], T. P. Resende[1], A. F. Araújo[1,4], M. Valente[1,2], J. Almeida[3], J. P. Martins[3], J. M. Santos[3], R. N. Barcia[3], P. Cruz[3], H. Cruz[3] and P. Pinto-do-Ó[1,2]

¶ These authors contributed equally to this work.

Supplementary Methods

UCX® characterization

Cells were characterized by their capacity to adhere to plastic in standard MSCs culture conditions. Surface marker expression was analyzed by flow cytometry in a Gallios imaging flow cytometer (Beckman Coulter Inc., CA, USA). Tryple Select-detached cells were passed through a 70µm filter (BD Falcon) and the single cell suspensions stained by 1 h incubation at 4°C with the antibodies in 2% (w/v) BSA (Sigma-Aldrich) and washed in PBS (Sigma-Aldrich). The following anti-human antibodies (Biolegend, Inc. unless stated otherwise) were used: conjugated phycoerythrin (PE)-CD105 (eBioscience), -CD90, conjugated allophycocyanin (APC)-CD73, conjugated PerCp/Cy5.5-CD14 and -CD45, conjugated fluorescein isothiocyanate (FITC)-CD34, conjugated pacific blue-CD19 and -HLA-DR. The isotype-matching control antibodies were: pacific blue-IgG1, -IgG2a, PerCp/Cy5.5-IgG1k, -IgG2a, PE-IgG1k, APC-IgG1k and FITC-IgG1k.

Immunohistochemistry

Immunostaining of heart sections for human nuclei detection was performed following antigen recovery, permeabilization with 0,2% Triton X-100 for 5 minute and 1 hour incubation in M.O.M. blocking solution (M.O.M.™ Immunodetection Kits, Vector Labs). Sections were incubated with primary antibody (MAB4383, mouse anti-human nuclei, Milipore), diluted 1:400 in the blocking solution, for 2 hours at RT. Thereafter, sections were incubated with biotin-MOM IgG secondary antibody (1:250) 15 min at RT and the signal was amplified by incubation with 488-Streptavidin (1:500, Invitrogen) 30 min, RT. Sections were then mounted using Fluoroshield containing DAPI (F6057, Sigma-Aldrich).

Gene array

RNA from UCX® cells cultivated in MSC-medium was isolated using the RNeasy Mini Kit, Qiagen, Hilden, Germany, following the protocols of the manufacturer. Nearly $5x10^6$ cells were collected after trypsinization. After cell lysis homogenization was performed by passing the lysate 5 times through a 20 gauge syringe and DNAse digestion was used to eliminate DNA contamination. Quality and integrity of the total RNA isolated was controlled on a bioanalyzer (Agilent Technologies; Waldbronn, Germany). 5 µg of total RNA was used for biotinylated target synthesis according to standard protocols supplied by the manufacturer (Affymetrix; Santa Clara, CA). Briefly, RNA was converted to dsDNA using 100 pmol of a T7T23V primer (Eurogentec; Seraing, Belgium) containing a T7 promoter. The cDNA was then used directly in an *in vitro* transcription reaction in the presence of biotinylated nucleotides. The concentration of biotin-labeled cDNA was determined by UV absorbance. For hybridization, 10 µg of each biotinylated

cDNA preparation were fragmented and placed in a hybridization cocktail containing also 4 biotinylated hybridization controls (BioB, BioC, BioD, and Cre) as recommended by the manufacturer. Samples were hybridized for 16 hours to Affymetrix Gene Chip HG_U133 Plus 2.0, representing about 47000 human transcripts. After hybridization the GeneChips were washed, stained with SA-PE and read using an Affymetrix GeneChip fluidic station and scanner. Analysis of microarray data [GEO:GSE51869] was performed using the Affymetrix Microarray Suite 5.0, and BRB Array Tools 4.2. All array experiments were normalized using RMA.

Supplementary Figures

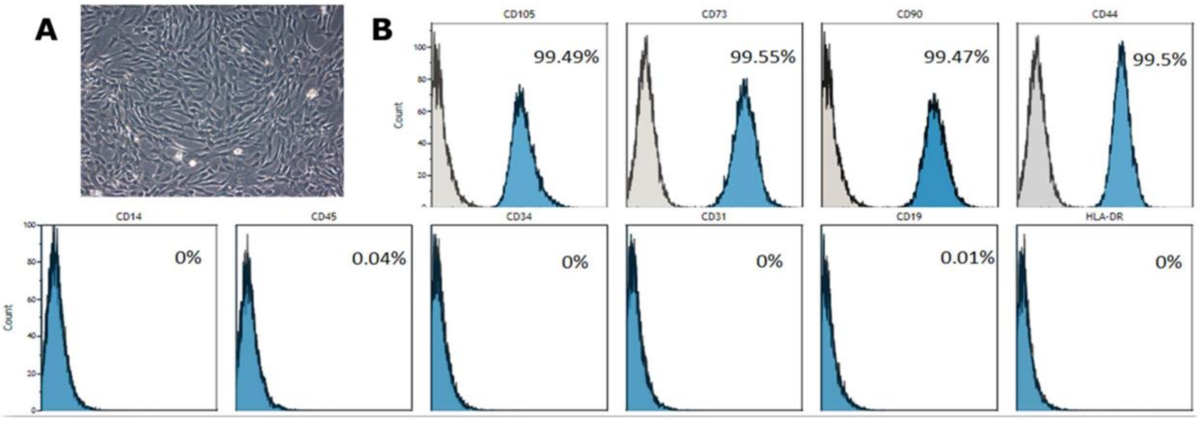

Supplemental Figure 1 – UCX® display a mesenchymal affiliated profile compliant with ISCT guidelines for the definition of MSC. (**A**) UCX® displayed plastic adherence and fibroblast-like morphology (100x).

(**B**) Flow cytometry analysis of cell surface markers revealed that over 95% of the cells expressed CD105, CD73, CD90 and CD44 and less than 2% of the cells expressed CD14, CD45, CD34, CD31, CD19 and HLA-DR (UCX® in blue and isotype control in light grey).

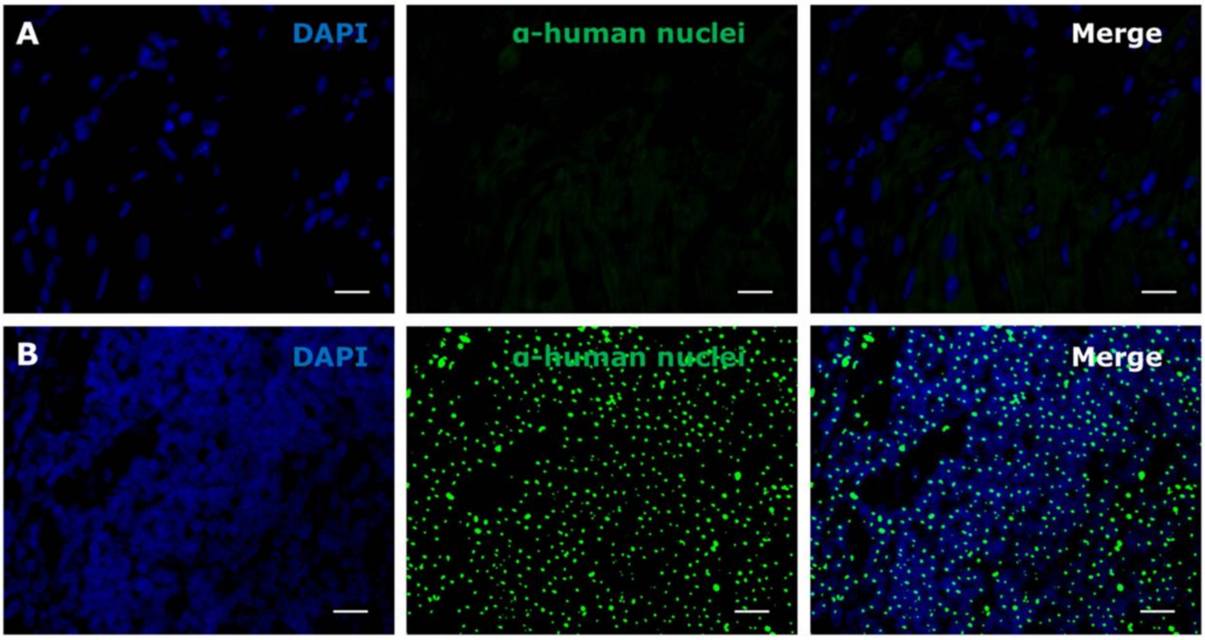

Supplemental Figure 2 – Fourteen-days following infarction, murine hearts transplanted with UCX® **(A)** do not display human nuclei indicating that the engraftment of transplanted cells of human origin did not occur. As a positive control, human muscle tissue was used **(B)**. Scale bar = 20 μm.

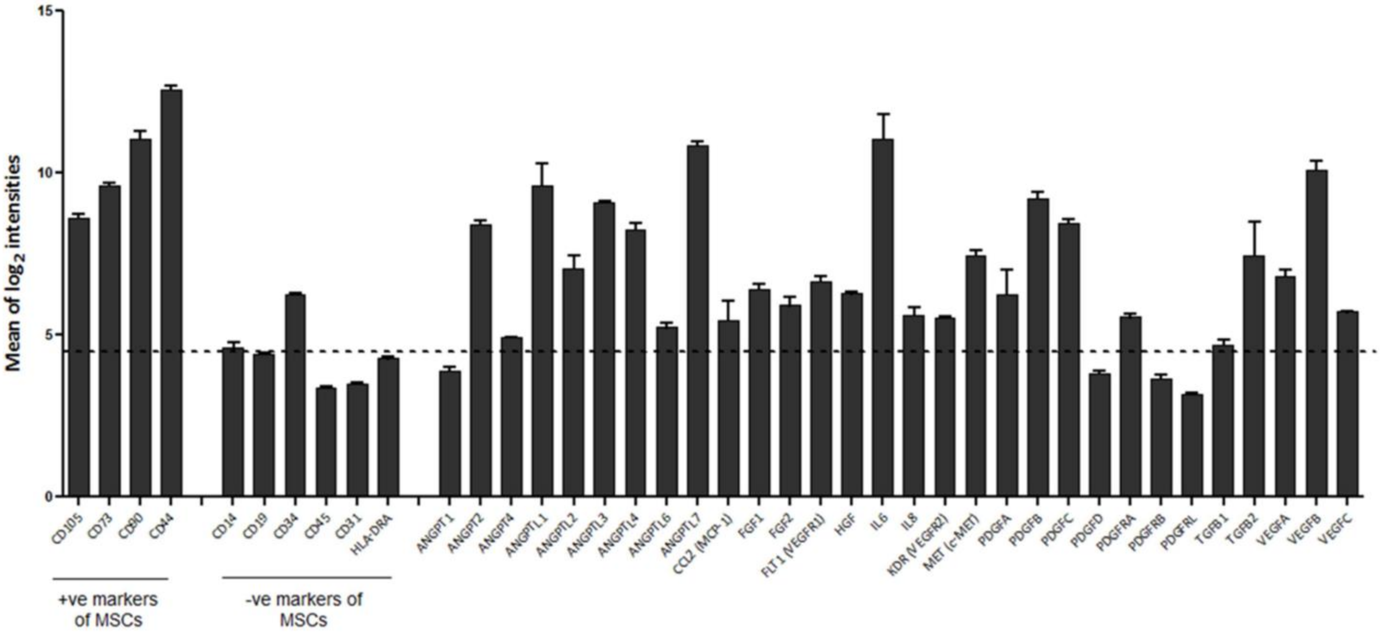

Supplemental Figure 3 – UCX® gene expression profile

Relative gene expression of known MSC-and angiogenic-related genes in UCX® derived from 3 different umbilical cords are represented as mean ± s.e.m. Dotted line was positioned on the average negative known markers of MSCs. Genes with relative expression values above this line were considered as up-regulated. Gene expression array of UCX® from 3 different umbilical cords relative to that of positive (CD105, CD73, CD90 and CD44) and negative (CD14, CD19, CD34, CD45, CD31 and HLA-DR) markers of MSCs reveal that several angiogenic factors (VEGF, angiopoietins, HGF, CmET, bFGF, TGF-β and PDGF-AB) are expressed in high levels, which could explain the observed paracrine effects.
